# RAD001 Enhances the Potency of BEZ235 to Inhibit mTOR Signaling and Tumor Growth

**DOI:** 10.1371/journal.pone.0048548

**Published:** 2012-11-14

**Authors:** Beat Nyfeler, Yan Chen, Xiaoyan Li, Maria Pinzon-Ortiz, Zuncai Wang, Anupama Reddy, Elina Pradhan, Rita Das, Joseph Lehár, Robert Schlegel, Peter M. Finan, Z. Alexander Cao, Leon O. Murphy, Alan Huang

**Affiliations:** 1 Developmental and Molecular Pathways, Novartis Institutes for BioMedical Research, Cambridge, Massachusetts, United States of America; 2 Oncology Translational Medicine, Novartis Institutes for BioMedical Research, Cambridge, Massachusetts, United States of America; University of Colorado School of Medicine, United States of America

## Abstract

The mammalian target of rapamycin (mTOR) is regulated by oncogenic growth factor signals and plays a pivotal role in controlling cellular metabolism, growth and survival. Everolimus (RAD001) is an allosteric mTOR inhibitor that has shown marked efficacy in certain cancers but is unable to completely inhibit mTOR activity. ATP-competitive mTOR inhibitors such as NVP-BEZ235 can block rapamycin-insensitive mTOR readouts and have entered clinical development as anti-cancer agents. Here, we show the degree to which RAD001 and BEZ235 can be synergistically combined to inhibit mTOR pathway activation, cell proliferation and tumor growth, both *in vitro* and *in vivo*. RAD001 and BEZ235 synergized in cancer lines representing different lineages and genetic backgrounds. Strong synergy is seen in neuronal, renal, breast, lung, and haematopoietic cancer cells harboring abnormalities in PTEN, VHL, LKB1, Her2, or KRAS. Critically, in the presence of RAD001, the mTOR-4EBP1 pathway and tumorigenesis can be fully inhibited using lower doses of BEZ235. This is relevant since RAD001 is relatively well tolerated in patients while the toxicity profiles of ATP-competitive mTOR inhibitors are currently unknown.

## Introduction

The mammalian target of rapamycin (mTOR) is a key regulator of cell growth, proliferation and cellular metabolism, and is activated downstream of several oncogenes and tumor suppressors. Amplification and activating mutations in *PIK3CA*, deletions and loss of function mutations in *PTEN*, *LKB1* or *TSC2* can all constitutively activate mTOR and its downstream signaling events [1], [2]. mTOR functions as part of at least two multi-protein complexes, mTORC1 and mTORC2. Both of these complexes have been shown to have important roles in tumorigenesis [3], [4]. While mTORC1 promotes cell growth by phosphorylating certain substrates including S6K1 and 4EBP1, mTORC2 is thought to promote cell survival primarily via the phosphorylation of Akt [5]. The high frequency of somatic and germline mutations resulting in mTOR pathway activation makes pharmacological inhibition of mTOR a promising therapeutic avenue for a variety of human cancers. Two main classes of mTOR inhibitors have been developed: allosteric inhibitors, typified by rapamycin and its derivatives (rapalogues); and ATP-competitive mTOR inhibitors [6]–[8].

Rapamycin (sirolimus) and its derivatives such as RAD001 (everolimus) or CCI-779 (temsirolimus) function along with their intracellular receptor FKBP12 as highly selective allosteric inhibitors of mTORC1. Binding of rapamycin to FKBP12 allows the complex to bind to mTOR which results in a conformational change in mTORC1 and inhibition of substrate phosphorylation [9]. Rapamycin-FKBP12 does not bind mTORC2 but prolonged rapamycin treatment has been shown to interfere with mTORC2 assembly [10]. RAD001 inhibits proliferation in a wide variety of tumor cell lines both *in vitro* and *in vivo* and has received FDA-approval for the treatment of a subset of cancer types including advanced renal carcinoma, pancreatic neuroendocrine cancers, and cancers associated with hereditary tuberous sclerosis, including subependymal giant cell astrocytomas and angiomyolipomas of the kidney.

ATP-competitive mTOR inhibitors directly target the mTOR kinase pocket. Two classes of inhibitors are in clinical development. BEZ235 [11] and PI-103 [12] are typical dual kinase inhibitors of mTOR and PI3K, while Ku-0063794, WYE-354, PP242, Torin1 and AZD8055 represent mTOR-selective kinase inhibitors [6]–[8]. ATP-competitive mTOR inhibitors of either class can target the catalytic site of both mTORC1 and mTORC2, and have revealed the existence of rapamycin-insensitive mTORC1 outputs such as the phosphorylation of 4EBP1 T37/46 or the induction of autophagy [13]–[18]. Such catalytic mTOR inhibitors show broad preclinical antitumor activity *in vitro* and *in vivo* and are currently undergoing clinical evaluation [6]–[8].

We previously observed that RAD001 and the catalytic mTOR inhibitor Ku-0063794 can act synergistically to target rapamycin-insensitive mTORC1 outputs such as cap-dependent translation and autophagy in SK-N-SH neuroblastoma cells [16]. Xu and colleagues [19] and Thomas and colleagues [20] have reported that the combination of RAD001 and BEZ235 has synergistic anticancer activity in non-small cell lung cancer and hepatocellular carcinoma, respectively. In the current study, we show that the synergistic combination of RAD001 and BEZ235 is not restricted to these two tumor types, but rather inhibits mTOR signaling, cell viability and tumorigenesis broadly in several different cancer lines, representing various lineages and genetic backgrounds. In the presence of RAD001, low doses of BEZ235 resulted in anticancer activity and gene expression changes very similar to those seen with high doses of BEZ235 as single agent. These data show that RAD001 enhances the potency of BEZ235 in a profoundly synergistic manner which is of clinical relevance given the need for effective therapeutic agents at the lowest dose possible.

## Materials and Methods

### Antibodies and inhibitors

Antibodies against total 4EBP1, phospho-4EBP1 T37/46, total S6K1, phospho-S6K1 T389, PARP and GAPDH were obtained from Cell Signaling Technology. The antibody against ß-actin was purchased from Ambion. NVP-RAD001 (Novartis), NVP-BEZ235 (Novartis), Ku-0063794 (Chemdea) and Staurosporin (Tocris Bioscience) were used with DMSO (Calbiochem) as solvent vehicle.

### Cell culture and cell viability assays

All cell lines were purchased from American Type Cell Collection and were cultured at 37°C in a 5% CO_2_ incubator. U-87 MG cells were maintained in MEM (Invitrogen #11095) complemented with 10% fetal bovine serum. NCI-H23, 786-O, KMS-11 and Pfeiffer cells were maintained in RPMI1640 (ATCC #30-2001) media complemented with 10% fetal bovine serum. SK-BR-3 cells were maintained in McCoy's 5A (ATCC # 30-2007) complemented with 10% fetal bovine serum. Cell viability was determined by measuring cellular ATP content using the CellTiter Glo luminescence assay (Promega). One day before drug addition, 1000 cells were plated into 384-well plates (Greiner) in 30 µl growth media. Cells were then incubated for 72 h with various concentrations of drugs or combinations before CellTiter Glo reagent was added to each well and luminescence recorded on an Envision plate reader (Perkin Elmer). Luminescence values were used to calculate the inhibition of cell viability relative to DMSO-treated cells (0% inhibition).

### Cytotoxicity xCELLigence assay

The xCELLigence system (Roche Applied Science) was used to assess the cell index of SK-BR-3 cells. xCELLigence measures electrical impedance across micro-electrodes integrated at the bottom of tissue culture E-plates which provides quantitative information about the biological status of the cells, including cell number, morphology and viability. One day before drug addition, SK-BR-3 cells were seeded in E-plates 96 at a density of 4500 cells per well in 80 µl growth media. Subsequently, 20 µl compound-containing growth media was added to each well. Each treatment was tested in triplicate and compared to cells treated with an equivalent volume of DMSO as control. Electric impedance was measured in 15-minute intervals from the time of plating until the end of the experiment. Cell index values, which is a dimensionless parameter derived as a relative change in measured electrical impedance to represent numbers of attached cells, were calculated from the electric impedance and plotted using the RTCA software provided by the vendor.

### Phospho-4EBP1 high-content assay

Phosphorylation of 4EBP1 was quantified with an immunofluorescence-based high-content assay. 3000 cells were plated into 384-well plates (Greiner), allowed to attach overnight, incubated with various concentrations of drugs or drug combinations, fixed with Mirsky's fixative (National Diagnostics), stained overnight using phospho-4EBP1 T37/46 antibody (Cell Signaling Technology, #2855), washed, stained with Cy5-conjugated goat-anti-rabbit IgG (Millipore) and Hoechst33342 (Invitrogen), imaged using the InCell Analyzer 1000 (GE Healthcare), and cellular fluorescence intensities were determined using the InCell Investigator software (GE Healthcare). Background fluorescence (cells stained without primary antibody) was subtracted, and inhibition of the phosphorylation signal was calculated relative to DMSO-treated cells (0% inhibition).

### Calculating of compound synergy

RAD001 was tested in combination with BEZ235 or Ku-0063794 using different dose matrices. Relative inhibition of 4EBP1 phosphorylation or cell viability was calculated for every dose combination. Using the Chalice software [21], the response of the combination was compared to its single agents, against the widely used Loewe model for drug-with-itself dose-additivity [22]. Excess inhibition compared to additivity can be plotted as a full dose-matrix chart to visualize the drug concentrations where synergies occur.

### Immunoblotting

Cell lysates were generated using RIPA buffer supplemented with protease inhibitor cocktail (Roche), cleared by centrifugation for 20 min at 13,000 rpm, and protein concentrations were quantified with the BCA TM protein assay kit (Pierce). Protein samples were boiled in SDS loading buffer, resolved on NuPage 4–12% Bis-Tris gels with MES or MOPS SDS running buffer (Invitrogen), and transferred to nitrocellulose membranes. The blots were blocked for 1 h at room temperature and probed with primary antibodies overnight at 4°C. Immobilized primary antibodies were visualized using either HRP-conjugated anti-rabbit IgG (Millipore) with enhanced chemiluminescence (Pierce), or IRDY800- and Alexa Fluor 700-conjugated anti-mouse and anti-rabbit IgG with the Li-COR ODYSSEY V1.2 scanning and editing system.

### Microarray analysis

SK-BR-3 cell cultures were treated with vehicle control, 5 nM RAD001, 20 nM or 500 nM BEZ235, or the combination of 5 nM RAD001 and 20 nM BEZ235 for 24 h. Total RNA was isolated using the QIAGEN total RNeasy Mini Kit and samples of 0.4 µg RNA were subjected to Affymetrix gene chip U133 plus 2.0 analysis. The microarray data were MAS5 normalized (Table S1), and probes with very low signal across all treatment conditions were filtered out (mean + SD<100). Differential analysis was performed by computing fold changes of treatment to baseline (vehicle control), and genes with a fold change >2 were defined to be differentially regulated. Pathways enriched in the differential genes were computed using Fisher's test (p-value<0.05) to test the significance of the overlap in the Gene Ontology (GO) biological processes database (www.geneontology.org). Significant GO terms (FDR p-value<0.25) in the same hierarchical branch were clustered, and the parent term from that cluster was selected as a representative label. All the microarray analysis was performed using the statistical package R (v2.10.1) and TIBCO Spotfire (v3.2.1).

### RAD001 and BEZ235 *in vivo* experiments

The *in vivo* efficacy of RAD001, BEZ235, or their combination was evaluated in U-87 MG and 786-O human tumor xenograft models, in accordance with institutional and Institutional Animal Care and Use Committee guidelines. U-87 MG cells were implanted subcutaneously at 5×10^6^ cells/mouse in female nude mice. Tumor-bearing mice were randomized into control and treatment groups when tumors reached ∼124 mm^3^ in size (Day 1) with a cohort size of 10 per group. BEZ235, RAD001, or their combination was given orally once a day for 4 weeks, and the pre-determined endpoint was a tumor volume of 1500 mm^3^. 786-O cells were implanted subcutaneously at 1×10^7^ cells/mouse in female nude mice. Tumor-bearing mice were randomized into control and treatment groups when tumors reached ∼144 mm^3^ in size (Day 1), with a cohort size of 10 per group. BEZ235, RAD001 or their combination was given orally once daily for 3 weeks, and the pre-determined endpoint was a tumor volume of 1000 mm^3^. Tumor volumes were quantified over time and calculated as follows: (tumor width)^2^×(tumor length)×0.5. The change in tumor volume was determined after 26 days for U-87 MG tumors and 22 days for 786-O tumors, and treatment over control (T/C) values were calculated as T/C (%) = (ΔT/ΔC)×100, for ΔT>0. T/T0 (%) = (ΔT/T0)×100, for ΔT<0; where ΔT is the change in mean tumor volume of treatment cohort from day 1 to endpoint, ΔC is the change of mean tumor volume of vehicle cohort from day 1 to endpoint, and T0 is the mean tumor volume of treatment cohort on day 1. The statistical significance of the difference was determined with the nonparametric Kruskal-Wallis test, with Dunn's multiple comparison test.

## Results

### RAD001 and BEZ235 synergistically inhibit mTOR signaling and tumorigenesis

**Figure 1:**
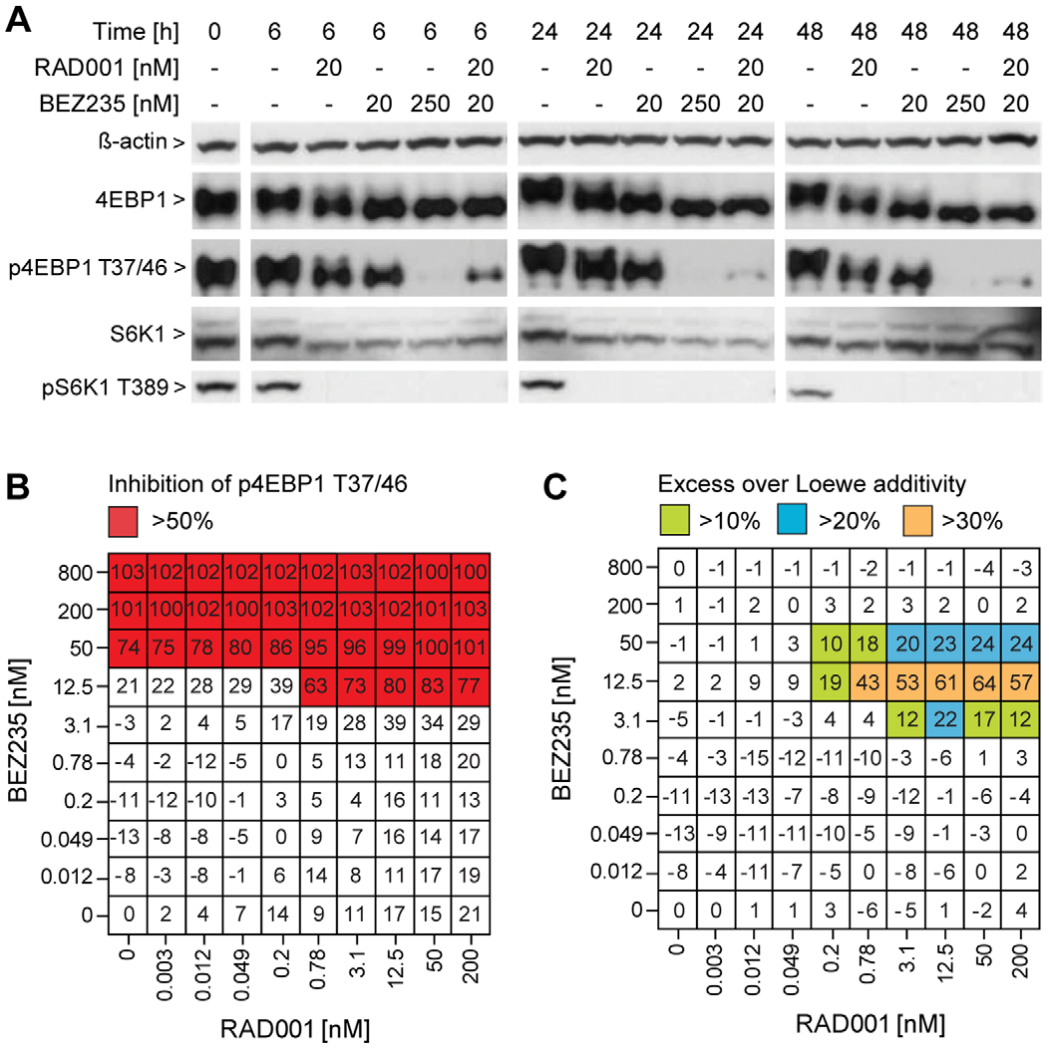
RAD001 and BEZ235 synergistically inhibit mTORC1 signaling in U-87 MG cells. U-87 MG (PTEN null glioblastoma) cells were treated for 6, 24 or 48 h with the indicated doses of RAD001 and/or BEZ235. After lysing the cells, endogenous proteins were visualized by western blot analysis (A). U-87 MG cells were treated for 24 h with different combinations of RAD001 and/or BEZ235, after which 4EBP1 T37/46 phosphorylation was quantified using high-content imaging and analysis (B). Data values show inhibition relative to DMSO-treated cells and represent the mean from 4 wells. Chalice software was used to calculate excess inhibition over Loewe additivity for each RAD001 and BEZ235 dose combination (C).

We chose the PTEN null glioblastoma U-87 MG cell line as a first model to explore the combination of RAD001 and BEZ235, and monitored mTOR pathway inhibition by western blot analysis. As single agent, 20 nM RAD001 completely inhibited S6K1 T389 phosphorylation with minimal effects on 4EBP1 T37/46 phosphorylation, while 250 nM BEZ235 robustly inhibited both S6K1 T389 and 4EBP1 T37/46 phosphorylation (Figure 1A). This is in line with the finding that ATP-competitive mTOR inhibitors can block rapamycin-insensitive mTOR outputs such as 4EBP1 T37/46 phosphorylation [13]–[18]. The low dose of 20 nM BEZ235 only inhibited S6K1 T389 phosphorylation but showed a robust effect on 4EBP1 T37/46 phosphorylation when combined with 20 nM RAD001, similar to the high dose of 250 nM BEZ235 as single agent (Figure 1A). This synergy was confirmed by testing RAD001 and BEZ235 in a 10×10 dose matrix using a phospho-4EBP1 T37/46 high-content assay (Figure 1B, left column and lowest row reflect single agent activities of BEZ235 and RAD001, respectively). With increasing amounts of RAD001, lower doses of BEZ235 were required to inhibit 4EBP1 T37/46 phosphorylation. The interaction of RAD001 and BEZ235 was synergistic and excess inhibition scores over Loewe additivity were observed for 3.1–50 nM BEZ235 when combined with more than 0.2 nM RAD001 (Figure 1C).

**Figure 2:**
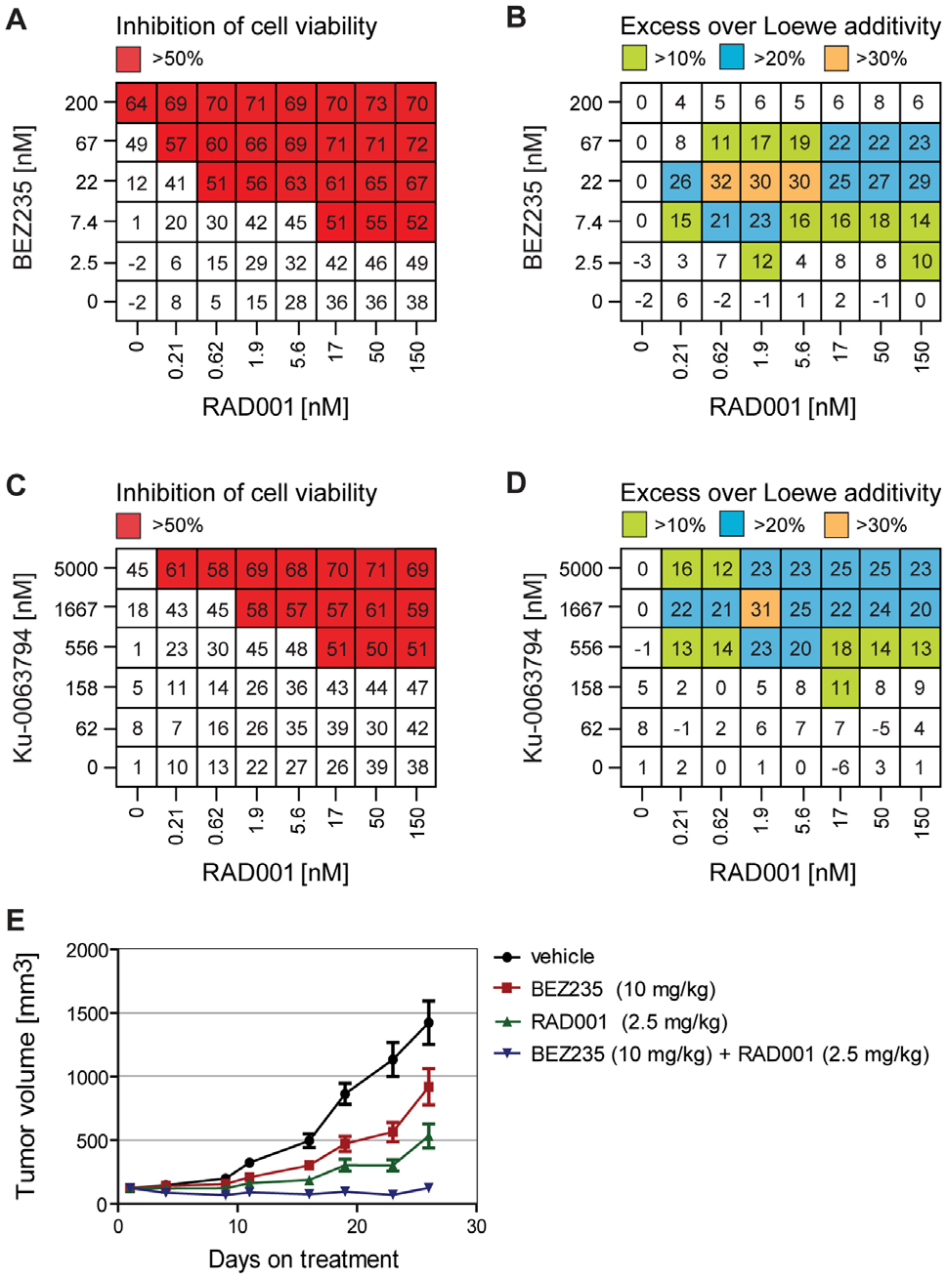
RAD001 and BEZ235 synergistically inhibit growth of U-87 MG cells *in vitro* and *in vivo*. U-87 MG cells (PTEN null glioblastoma) were treated for 72 h with different doses of RAD001 and/or BEZ235, inhibition of cell viability was measured using the CellTiter-Glo assay (A), and Chalice software was used to calculate excess inhibition over Loewe additivity for each RAD001 and BEZ235 dose combination (B). U-87 MG cells were treated for 72 h with different doses of RAD001 and/or Ku-0063794, inhibition was measured using CellTiter-Glo (C), and Chalice was used to calculate excess over Loewe additivity (D). Data values represent the mean from 3 wells. Mice bearing U-87 MG xenografts were dosed daily for 4 weeks with RAD001, BEZ235, or the combination of the two compounds. Mean tumor volumes were measured and are plotted as a function of time (E).

To determine whether the synergistic inhibition of mTOR signaling led to reduced cell viability, the combination of RAD001 and BEZ235 was tested in a 3-day assay using CellTiter Glo as read out. As single agents, the highest doses of 150 nM RAD001 and 200 nM BEZ235 reduced cell viability 38% and 64%, respectively (Figure 2A). The combination of 150 nM RAD001 and 200 nM BEZ235 did not have a significant additive effect and reduced cell viability 70%, similar to 200 nM BEZ235 as single agent. However, as observed for 4EBP1 T37/46 phosphorylation (Figure 1B), lower doses of BEZ235 were required to reduce cell viability when combined with RAD001 (Figure 2A), and synergistic values were observed in the Loewe additivity model (Figure 2B). Since BEZ235 has antagonistic activity against both PI3K and mTOR kinases [11], we tested if RAD001 could inhibit cell viability in a synergistic manner if combined with an mTOR-selective kinase inhibitor such as Ku-0063794 [15]. Like for BEZ235, lower doses of Ku-0063794 were required to reduce cell viability when combined with RAD001 (Figure 2C), and over 20% excess inhibition over Loewe additivity were observed for doses of 556 nM up to 5000 nM Ku-0063794 (Figure 2D). This suggests that the mTOR inhibitory activity of BEZ235 largely accounts for the synergistic interaction with RAD001.

The efficacy of combining RAD001 and BEZ235 was next tested in the U-87 MG xenograft model. Tumor-bearing mice were treated for 4 weeks daily with 2.5 mg/kg RAD001, 10 mg/kg BEZ235, or the combination of the two agents. Inhibition of tumor growth with RAD001 and BEZ235 monotherapy was not significant with T/C values of 31% and 59%, respectively (Figure 2E). In contrast, the combination of RAD001 and BEZ235 significantly (p<0.001) reduced tumor growth with a T/C of 0% (Figure 2E).

### RAD001 and BEZ235 show synergistic anti-cancer activity in cancer lines of different lineages and genetic backgrounds

**Figure 3:**
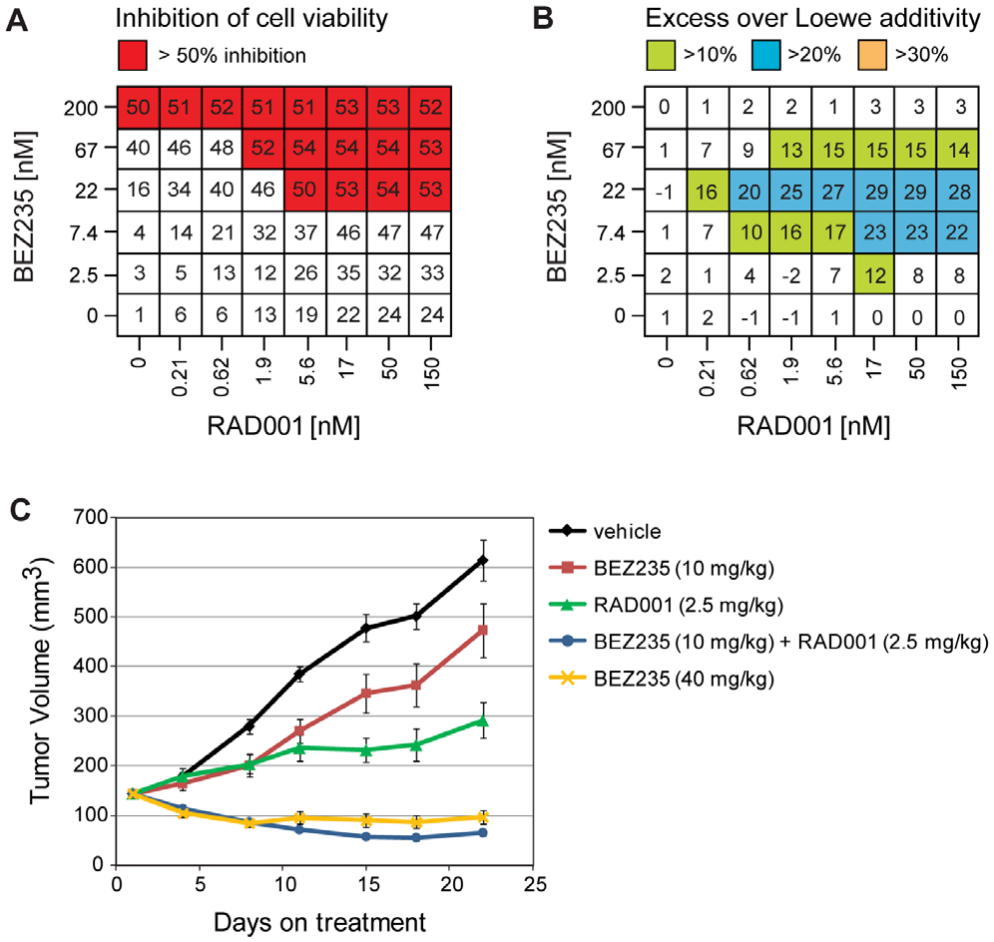
RAD001 and BEZ235 synergistically inhibit growth of 786-O cells *in vitro* and *in vivo*. 786-O cells (PTEN and VHL-deficient renal cell carcinoma) were treated for 72 h with different doses of RAD001 and/or BEZ235, inhibition of cell viability was measured using CellTiter-Glo (A), and Chalice software was used to calculate excess inhibition over Loewe additivity for each RAD001 and/or BEZ235 dose combination (B). Data values represent the mean from 3 wells. Mice bearing 786-O xenografts were dosed daily for 3 weeks with RAD001, BEZ235, or the combination of the two compounds. Mean tumor volumes were measured and are plotted as a function of time (C).

We next explored the combination of RAD001 and BEZ235 in cancer lines representing different genetic backgrounds and lineages. In the PTEN and VHL-deficient renal cell carcinoma line 786-O, RAD001 and BEZ235 strongly synergized *in vitro* and reduced cell viability with excess inhibition over Loewe additivity when 2.5–67 nM BEZ235 were combined with RAD001 (Figure 3A and 3B). *In vivo*, 2.5 mg/kg RAD001 and 10 mg/kg BEZ235 inhibited tumor growth as single agents with a T/C of 31% and 70%, respectively (Figure 3C). The combination of 2.5 mg/kg RAD001 and 10 mg/kg BEZ235 was significantly more efficacious with a T/T0 of −54% (p<0.001), similar to the high dose of 40 mg/kg BEZ235 with a T/T0 of −32% (p<0.001). Hence, the combination of RAD001 and BEZ235 has synergistic inhibitory activity on cell viability *in vitro* and tumor growth *in vivo* in the 786-O tumor model.

**Figure 4:**
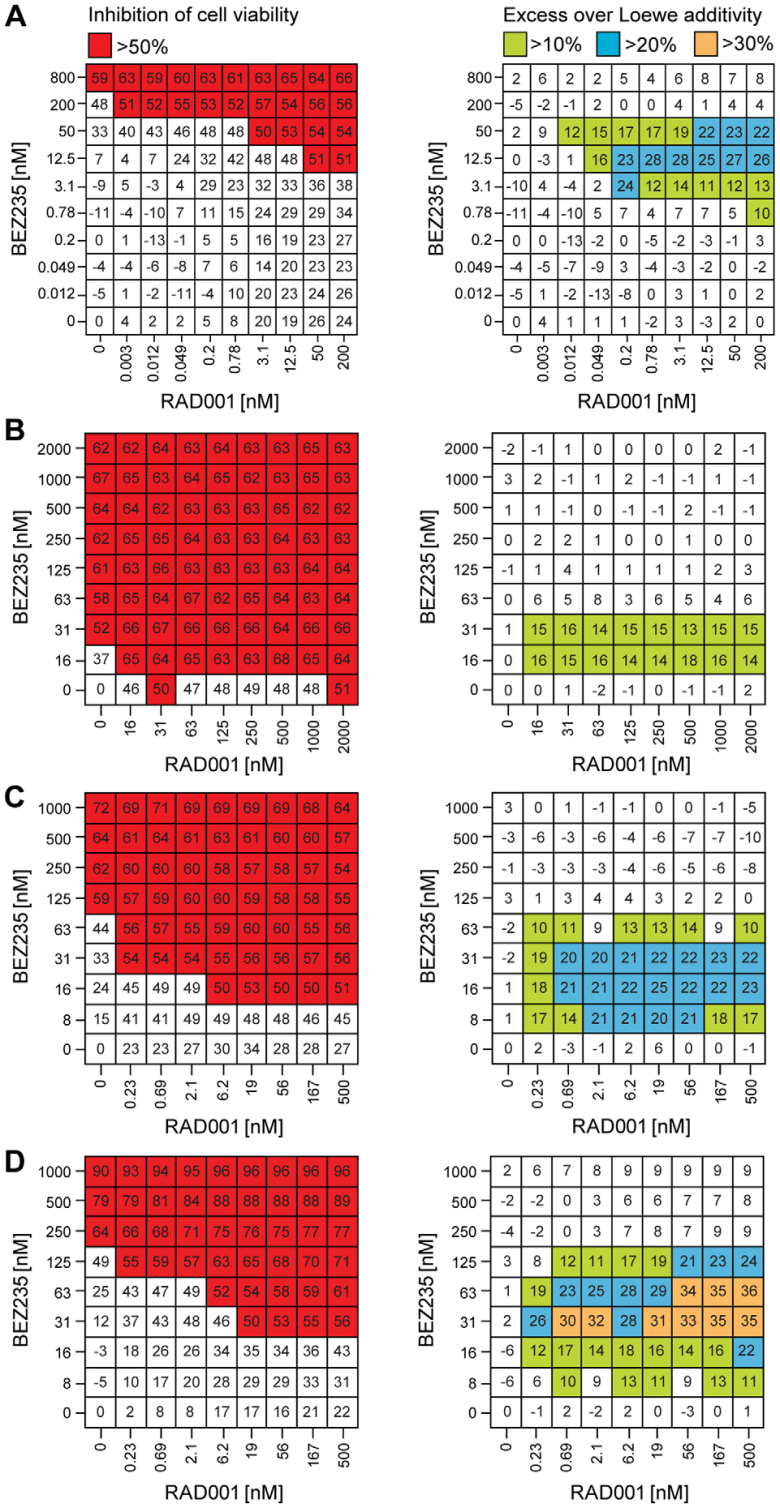
RAD001 and BEZ235 synergistically inhibit cell viability in cancer lines of various lineages and genetic backgrounds. KRAS/LKB1 mutant non-small cell lung cancer NCI-H23 (A), HER2-amplified SK-BR-3 breast cancer SK-BR-3 (B), multiple myeloma KMS11 (C) and diffuse large B-cell lymphoma Pfeiffer cells (D) were treated for 72 h with different doses of RAD001 and/or BEZ235, inhibition of cell viability was measured using CellTiter-Glo, and Chalice was used to calculate excess over Loewe additivity. Data values represent the mean from 3 wells.

We next monitored cell viability and excess inhibition over Loewe additivity in additional cancer lines including the non-small cell lung cancer line NCI-H23 harboring mutations in *KRAS* and *LKB1* (Figure 4A), the HER2-amplified SK-BR-3 breast cancer line (Figure 4B), the multiple myeloma cancer line KMS11 (Figure 4C) and the diffuse large B-cell lymphoma line Pfeiffer (Figure 4D). In all lines tested, RAD001 robustly enhanced the potency of BEZ235 and lowered the dose required to inhibit cell viability with synergistic values in excess of the Loewe additivity model. These results clearly demonstrate that RAD001 and BEZ235 have broad synergistic activity in several cancer models representing different lineages and genetic backgrounds.

### RAD001 with BEZ235 induces cell death in a *HER2*-amplified breast cancer model

**Figure 5:**
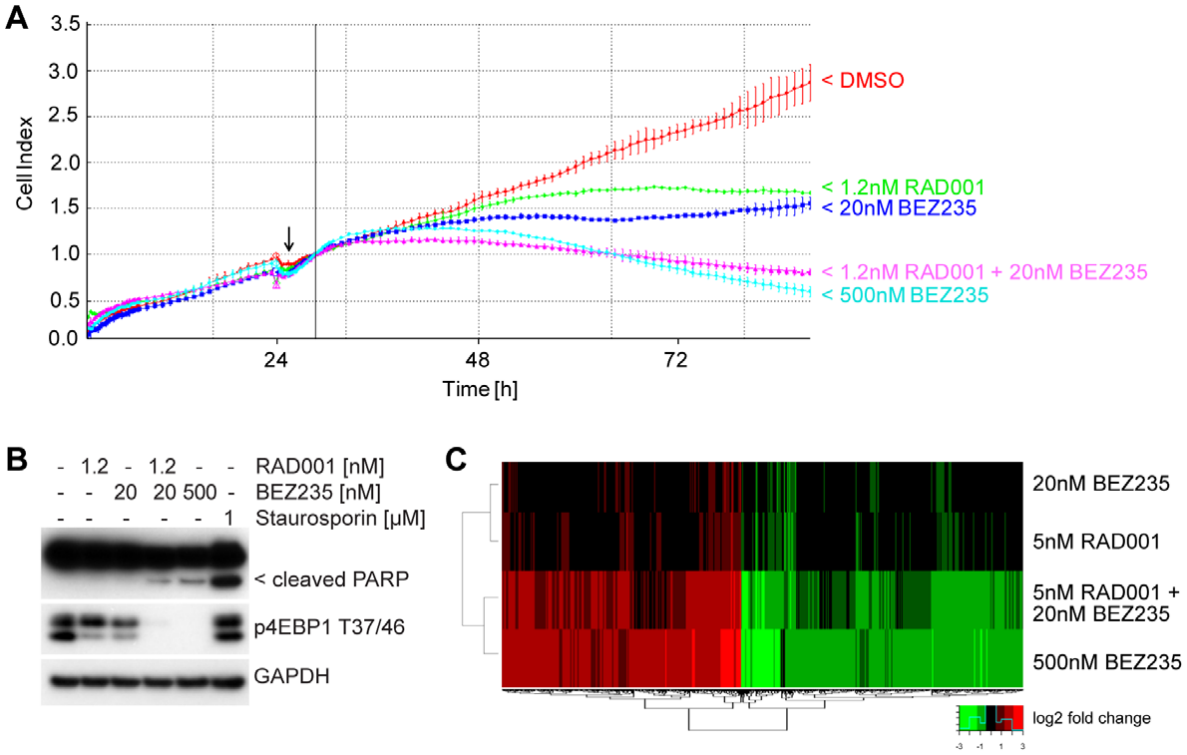
The combination of RAD001 and BEZ235 can induce cell death in SK-BR-3 cells. SK-BR-3 cells were treated with RAD001 and/or BEZ235 at the indicated concentrations and electric impedance was measured every 15 minutes with the xCELLigence system (A). Cell index values were calculated and plotted as a function of time. The time of compound addition is indicated with an arrow, and the continuous vertical line indicates the normalization point. Each data point represents the average of 3 wells ±1 SD. SK-BR-3 cells were treated for 48 h with the indicated doses of RAD001 and/or BEZ235, or for 3 h with Staurosporin. Cells were lysed and endogenous proteins visualized by western blot analysis (B). SK-BR-3 cells were treated for 24 h with the indicated doses of RAD001 and/or BEZ235. Gene expression changes were quantified by microarray analysis, computed as log2 relative ratios (treatment/vehicle control), and subjected to hierarchical clustering along the gene probe axis (C).

In *HER2*-amplified breast cancer cells such as SK-BR-3, BEZ235 has been shown to induce cell death while rapalogues only achieved growth arrest [11], [23]. The combination of RAD001 and BEZ235 clearly synergized in inhibiting cell viability in SK-BR-3 cells when assayed by CellTiter Glo (Figure 4B).To investigate whether the combination of RAD001 and a low dose of BEZ235 could actually induce cell death and regression, SK-BR-3 cell numbers were monitored in real-time using the xCELLigence system. Low doses of either 1.2 nM RAD001 or 20 nM BEZ235 clearly inhibited cell proliferation, but as single agent only the high dose of 500 nM BEZ235 was able to regress the cell index (Figure 5A). The combination of the low doses of 1.2 nM RAD001 and 20 nM BEZ235 resulted in an almost identical growth curve as the high dose of 500 nM BEZ235, indicating a regression of cell numbers. In line with the induction of cell death, 500 nM BEZ235 as well as the combination of 1.2 nM RAD001 and 20 nM BEZ235 resulted in PARP cleavage (Figure 5B). To determine whether the activity of the combination was likely achieved through pathway regulation, gene expression changes induced by RAD001 and BEZ235, either as single agents or in combination, were analyzed through gene expression profiling. Relative gene expression changes were computed for each treatment condition and then evaluated by hierarchical clustering (Figure 5C). While 5 nM RAD001 and 20 nM BEZ235 resulted in few gene expression changes as single agents, the combination of 5 nM RAD001 and 20 nM BEZ235 up-regulated 373 genes and down-regulated 429 genes more than two-fold (Table S2). These gene expression changes correlated well with the effects of the high dose of 500 nM BEZ235 (Pearson's R = 0.88) with a significant overlap of 694 genes (Fisher's p-value<10^−16^). At the pathway level (GO biological processes), the combination of RAD001 and BEZ235 predominantly regulated cell cycle-related pathways (data not shown). These results suggest that the combination of RAD001 and a low dose of BEZ235 can induce cell death in SK-BR-3 cells with gene expression changes that resemble those seen with the high dose of BEZ235 alone.

## Discussion

Inhibition of oncogenic signaling with targeted small molecule inhibitors is a powerful therapeutic approach to treat molecularly-driven tumors. Such inhibitors can be efficacious as single agents, but improved anti-tumor activity can often be achieved by combining with other cancer therapeutics [22]. For example, enhanced anti-tumor activity was reported by inhibiting two parallel oncogenic pathways by combining PI3K and MEK inhibitors [24]. Another example is the combination of allosteric and ATP-competitive inhibitors against Bcr-Abl which maximizes target inhibition and reduces the appearance of resistance [25]. Two previous reports [19], [20] and this current study illustrate that RAD001 and BEZ235 can be combined to synergistically inhibit mTOR signaling and tumor growth. Our observations suggest that lower doses of ATP-competitive mTOR inhibitors can achieve full target inhibition when combined with an allosteric mTOR inhibitor such as RAD001.

As a single agent, RAD001 completely inhibits the S6K1 effector pathway downstream of mTORC1 but only partially inhibits 4EBP1 T37/46 phosphorylation and cell proliferation [9], [16], [17]. On the other hand, a high dose of the ATP-competitive dual mTOR and PI3K inhibitor BEZ235 blocks both the S6K1 and 4EBP1 effector pathway and robustly affects cell proliferation with the induction of cell death in certain tumor models [11], [23]. Our new data shows that the combination with RAD001 does not enhance the maximal efficacy of BEZ235 but results in a consistent increase in its potency *in vitro* as well as *in vivo*. This is supported by the microarray analysis showing that the combination of RAD001 and a low dose of BEZ235 results in gene expression changes very similar to the response to a high dose of BEZ235 as a single agent (Figure 5C). This suggests that the combination with RAD001 allows BEZ235 to reach its inhibitory activity at a lower concentration.

We hypothesize that the binding of the FKBP12-RAD001 complex changes the conformation of mTORC1 in a way that its catalytic domain has an altered affinity for ATP-competitive inhibitors or is more accessible. Alternatively, binding of FKBP12-RAD001 might reduce the number of fully functional mTORC1 molecules thereby lowering the required dose to fully inhibit the remaining mTORC1 activity. The synergistic effect of RAD001 and BEZ235 is likely due to the action of both drugs on mTOR resulting in maximal kinase inhibition since synergy was also observed with the mTOR-selective kinase inhibitor Ku-0063794 [15], [19], [20]. In support of this, the combination of RAD001 and BEZ235 synergistically inhibited 4EBP1 T37/46 phosphorylation (Figures 1B and 1C), a read out very proximal to mTORC1. Importantly, the synergistic effects of RAD001 and BEZ235 were observed in several different cancer models representing different lineages and genetic backgrounds, suggesting that the synergistic effects are ubiquitous.

While RAD001 is relatively well tolerated in patients, its efficacy in the clinic is restricted to certain tumor indications. One potential caveat is the inability of rapalogues to inhibit mTORC2 or the rapamycin-insensitive outputs downstream of mTORC1 [13]–[18]. BEZ235 and other ATP-competitive mTOR inhibitors are able to fully suppress all known mTORC1 and mTORC2 functions but their clinical toxicity profiles are currently unknown. By combining RAD001 with a low dose of BEZ235, this study opens the possibility for effective target inhibition at lower doses. It will be interesting to see if this synergy can be translated into the clinic and if the interaction of allosteric and ATP-competitive inhibitors is a phenomenon which can be applied more broadly to other targeted therapies.

## Supporting Information

Table S1 **MAS5 normalized microarray data.** SK-BR-3 cells were treated with vehicle control, 5 nM RAD001, 20 nM or 500 nM BEZ235, or the combination of 5 nM RAD001 and 20 nM BEZ235 for 24 h. Total RNA was isolated and analyzed using Affymetrix gene chip U133 plus 2.0 analysis. MAS5-normalized data is shown.
(XLSX)

Table S2 **List of differentially regulated genes.** SK-BR-3 cells were treated with vehicle control, 5 nM RAD001, 20 nM or 500 nM BEZ235, or the combination of 5 nM RAD001 and 20 nM BEZ235 for 24 h. Total RNA was isolated and analyzed using Affymetrix gene chip U133 plus 2.0 analysis. Genes which are up-regulated or down-regulated more than two-fold upon treatment with the combination of 5 nM RAD001 and 20 nM BEZ235 are shown.
(XLS)
